# 

**Published:** 1885-09

**Authors:** 


					﻿SBPnMER,' 1885. [No. 3.
' . -
BW TW T ¥T"V V
AlMlXali b
...	>	Tef-J	!C'-A
ISsSk	L ~
y D s i j;k	gi i R N |t
ll®S^ '	1	f **.
?1Se
/Cr^Fxsoy-'
a'h/m'///>//'<//.• t .’.0>> #/ } >>tr in A h'ttw ;	> >pitCf-ftl >
EiMF.vr I’O.V HUECK Al AX V, PRIXTEH, A irSTlX/TEXA' ~
——.—  -. 
				

